# The Value of Radiation Therapists in Online Adaptive Radiotherapy

**DOI:** 10.1002/jmrs.70020

**Published:** 2025-08-28

**Authors:** Meegan Shepherd, Jackie Yim, Alexandra Turk, Leigh Ambrose, Alexander Podreka, John Atyeo

**Affiliations:** ^1^ Northern Sydney Cancer Centre Royal North Shore Hospital St Leonards New South Wales Australia; ^2^ Monash University Clayton Victoria Australia; ^3^ Centre for Health Economics Research and Evaluation, Faculty of Health University of Technology Sydney Sydney Australia

## Abstract

Value‐based health care (VBHC) is an evolving paradigm in healthcare that prioritises patient outcomes relative to the costs incurred. VBHC emphasises efficiency, quality, and patient satisfaction as metrics and determinants of healthcare. A VBHC approach is becoming increasingly significant as healthcare systems worldwide grapple with rising costs, staff shortages, and the ongoing need for improved patient care. Online adaptive radiotherapy (oART) is a promising innovation in radiation oncology, allowing for the adaptation of radiation treatment plans to account for daily anatomical changes. This innovation has the potential to improve patient outcomes; however, it requires investment in the technology, manpower, and training to deliver an adaptive radiotherapy service. This commentary aims to explore the role of radiation therapists (RTs) in oART, using RT‐led workflows within the context of the broader models of care that involve radiation oncologists and multidisciplinary teams. This commentary also aims to highlight the potential benefits and challenges of adopting an RT‐led approach within VBHC principles, focusing on key themes such as treatment accuracy, patient satisfaction, training, and cost implications.

## Graphical abstract

Value‐based health care (VBHC) prioritises patient outcomes relative to costs, emphasising efficiency, quality and patient satisfaction. Online adaptive radiotherapy (oART) is an emerging innovation in radiation oncology that enhances treatment accuracy and delivery, but requires investment in technology, training and staffing. This commentary explores the role of radiation therapists (RTs) in oART, highlighting the benefits and challenges of an RT‐led approach within VBHC principles, including treatment precision, patient satisfaction and cost implications.

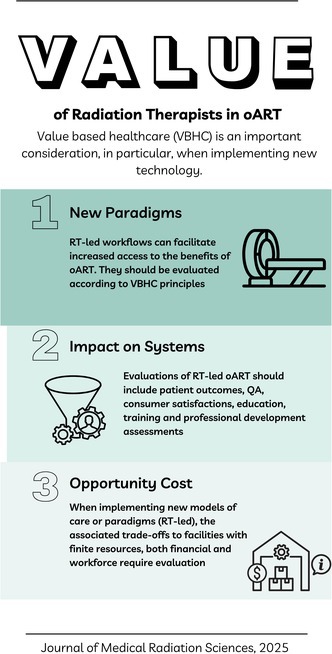

## 1 Background

Value‐based health care (VBHC) is a healthcare delivery model that emphasises maximising patient outcomes while minimising costs [1, 2, 3, 4]. Traditionally, fee‐for‐service models have incentivised the quantity of care provided; however, in VBHC, the focus is on the quality and efficiency of care [3]. By aligning financial incentives with patient health outcomes and preferences, this model encourages healthcare providers to adopt practices that enhance the effectiveness and efficiency of care delivery [3].

Teisberg et al. [4] define VBHC as care that is organised around the patient's medical condition, across the full cycle of care, with success measured by health outcomes that matter to patients relative to the cost of achieving them. In the context of radiotherapy, Leivens et al. [2] adapted this framework to highlight the importance of evidenced‐based treatment selection, resource‐efficient planning, and the reduction of unnecessary variation. Key VBHC principles in radiotherapy also include delivering the right care to the right patient at the right time, ensuring appropriateness, timeliness, and equitable access [2, 3, 4].

Radiotherapy is a major modality for cancer treatment, precisely targeting radiation to kill or damage cancer cells [5]. Technological advances have significantly improved the accuracy and effectiveness of radiotherapy, enabling improved dose to the target while sparing key organs at risk (OAR) and surrounding healthy tissue [6]. Adaptive radiotherapy (ART) is a practical solution to address changes in the patient's anatomy or tumour size, shape, and location that occur during radiotherapy [7]. Treatment plan adaptability is crucial in responding to the dynamic nature of anatomical variation, ensuring that the radiation dose remains carefully targeted throughout a treatment course [8].

Online adaptive radiotherapy (oART) represents a novel innovation in ART, utilising advanced imaging and real‐time image analysis to modify the treatment plan during each fraction [9]. This method enhances the precision of radiation delivery, accommodating anatomical changes such as tumour shrinkage, changes in body habitus, or internal organ movement [7]. The potential benefits of oART include improved targeting accuracy, target dose escalation or de‐escalation, reduced target margins, organ sparing, hypo‐fractionation, reduced interventions, and the ability to personalise treatment plans for each patient's unique daily situation [8]. By integrating continuous feedback and daily adaptive planning, oART holds promise for achieving better clinical outcomes and enhancing the overall effectiveness and value of radiotherapy [10].

## 2 The Traditional Multidisciplinary Team Approach

The traditional multidisciplinary team (MDT) approach in radiotherapy involves a coordinated effort, with each discipline contributing their expertise to deliver comprehensive cancer care [11]. The core team typically comprises radiation oncologists (RO), medical physicists (MP), RTs, and nurses, with additional input from allied health professionals such as speech pathologists and dietitians, and other specialists as needed [12]. While there may be some variation in role definition across global departments, each team member is essential to the radiotherapy process: ROs oversee patient prescribing, contouring, plan approval, and overall management of the patients treatment; MPs ensure the precision and safety of the radiation plan and equipment; RTs (or in some departments therapeutic radiographers and/or dosimetrists) complete image registration, some organ contouring, design the treatment plan, and work directly with patients to administer and monitor their treatment progress [13].

The patient workflow in the MDT approach is highly collaborative, a hallmark in the field of radiation oncology and necessary when implementing oART [14]. oART implementation is often guided by conventional treatment planning roles and responsibilities, with several ‘hand‐off’ points to various MDT members at the console [13]. For example, RTs handing off to ROs for target contour review or edit, and RTs to MPs for evaluation of treatment plan quality and deliverability. The strength of the oART MDT approach lies in the comprehensive expertise brought together to deliver each treatment fraction [8]. The MDT input fosters a holistic view of healthcare expertise, integrating knowledge and experience from each specialty to optimise daily treatment accuracy [11]. This approach also facilitates robust quality assurance (QA) and safety protocols, as multiple professionals review and scrutinise the daily treatment plan and its delivery.

**FIGURE 1:**
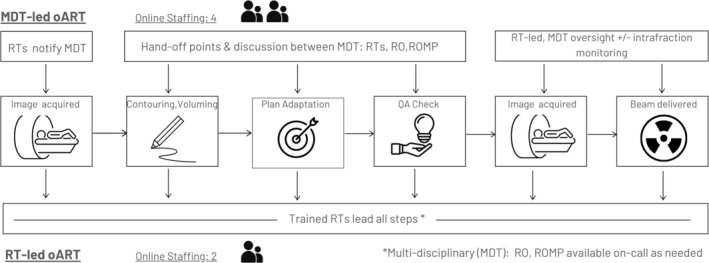
Comparison between traditional and multi‐disciplinary (MDT)‐led and radiation therapist (RT)‐led model of care workflows for online adaptive radiotherapy (oART). RO, radiation oncologist; ROMP, radiation oncology medical physicist; RT, radiation therapist.

Figure 1 and Table 1 compare the MDT‐led and RT‐led oART models, highlighting differences in roles, efficiency, scalability, and patient‐facing outcomes. These tools help to visualise how the RT‐led model may offer advantages in reducing time on bed and subsequent intra‐fraction motion, improving cost‐efficiency, and increasing scalability in workforce‐limited settings.

**TABLE 1:** Comparison of MDT‐led and RT‐led oART workflows with value based considerations.

Component	MDT‐led oART	RT‐led oART	Value based considerations
Image Acquisition	RT acquires image	RT acquires image, reviews, contours^a^, adapts plan^a^ and delivers treatment	Time saving, immediate imaging
Image Review	RT, ROMP reviews		Reduced MDT coordination
Contouring	RT, RO contours		Reduced cost, faster workflow
Plan Adaptation	RT, RO, ROMP reviews		Increased access, reduced resource dependency
QA Check	ROMP reviews		Streamlining process in favour of offline QA
Image Acquisition	RT, RO, ROMP reviews		MDT prioritising other clinical tasks
Treatment Delivery	RT delivers treatment		Shorter fraction duration, reduced intrafraction motion

Abbreviations: MDT, multi‐disciplinary team; RO, radiation oncologist; ROMP, radiation oncology medical physicist; RT, radiation therapist.

^a^ Steps or decisions are supported by AI, clinical decision aids, offline MDT availability via ‘on‐call’ arrangements, and/or patient specific handover documentation to ensure safety and continuity of care.

The MDT approach is not without its challenges. One significant issue is the potential for delays or inability to deliver oART due to the need for extensive coordination and consensus among team members present for each fraction [14]. The availability of various team members can delay the oART process, hinder decision‐making, or, in some instances, restrict or prevent its use [15]. Time is a key factor in oART, whereby inefficiency can impact intra‐fraction motion [14]. Another challenge is the higher cost associated with involving specialists and advanced technological resources, which may impact the decision to invest in healthcare innovation, particularly in rural centres [16]. An important study by McComas et al. [17] reviewed a time‐driven activity‐based costing for pelvis oART treatments. They found that with the MDT present, the average minimum cost per adapted fraction was $103.58 (USD). However, they did not investigate an RT‐led approach for oART. Understandably, in such a workflow, these costs would significantly decrease if the full MDT is not present. However, these costs may be transferred to training, education, or in post‐study QA or peer review [18]. Notably, in the McComas study, the value of oART was evident, with oART achieving significant dosimetric improvements compared with non‐adaptive plans, with reported mean target improvements of up to 16% in target V100% in favour of the adapted plan [17]. The clinical relevance of adaptive dosimetric improvements should be an area for future research relative to VBHC principles across various tumour indications. In addition to assessing the impact on individual patients, it will be an important endeavour for VBHC principles to evaluate the value versus cost of each of the dedicated oART systems for the broader cancer patient community.

## 3 Radiation Therapist‐Led oART Workflow

The RT‐led model for oART represents a significant shift in the traditional radiotherapy approach, highlighting the pivotal role of RTs in the delivery of oART. In this model, RTs take on extended responsibilities, including the use of advanced imaging technologies, online re‐contouring, adaptation of treatment plans, and online QA, ensuring the precise delivery of daily adapted radiation doses [15, 19, 20].

They are responsible for acquiring high quality imaging data such as Magnetic Resonance Imaging (MRI), interpretation of synthetic computed tomography (CT) generation, assessing anatomical changes, and adapting treatment plans accordingly, all while the patient is on the treatment couch [21]. This process begins with the patient undergoing daily imaging scans, such as MRI or cone‐beam CT (CBCT), to capture the current state of the tumour and surrounding organs. RTs are then presented with a workspace to adjust contours, some AI derived. The RTs evaluate contour accuracy and make edits focusing on key OARs and targets, whilst comparing the daily images with the original planning scan, identifying any significant changes that might affect the treatment plan [22]. The decision‐making process in oART is highly dynamic. RTs are able to modify treatment plans in real time, which requires a thorough understanding of oART protocols, effects of daily variations on medical physics principles, CT and MRI anatomy, and advanced imaging techniques. RTs must be proficient in quickly interpreting imaging data, adjusting treatment contours, and verifying the accuracy of the adapted plan before administering and monitoring it [23].

One of the primary advantages of the RT‐led model is increased access and efficiency. By empowering RTs to make daily adjustments, the need for frequent MDT attendance is reduced, lowering the costs associated with daily adaptations and accelerating the treatment process [24]. In the Royal Marsden study, by Adair‐Smith et al. (2023), oART contouring time was similar during pelvis fractions and mostly shorter for trained RTs than an RO‐led service. This theme has been demonstrated in other publications [25] including in an advanced practice RT (APRT) setting [26]. The literature hypothesises that dedicated time and personnel on the adaptive treatment unit may be important for familiarisation and efficiency of oART tools, which can unlock improvements in treatment outcomes and the reduction of risk and side effects, enhancing overall patient care [24, 27]. Understandably, at present, the oART process requires more time on the machine compared to daily IGRT; however, with advancements in technology speed and other factors, the timing for oART and IGRT may become largely similar, depending on the specific workflow and image guidance platform used.

The RT‐led model has the potential to deliver oART treatments without the RO attending. However, an RO may still be ‘on‐call’ for the adaptive fraction [13]. Comparing traditional MDT approaches and RT‐led oART models reveals significant insights into their effectiveness and areas for improvement. The MDT approach, with its comprehensive expertise and collaborative decision making, ensures thorough planning and high standards of care. However, it is often hampered by coordination delays and higher costs (initial and ongoing investment). Whereas the RT‐led oART model offers increased efficiency and has the potential for more timely adaptive treatments, fewer interventions and improved access. This model, however, requires substantial investment in technology, training and continuing professional development to ensure RTs are adequately equipped to manage the complex demands of the RT‐led oART model of care. This model also allows ROs to attend to other radiotherapy QA, training tasks or patient facing appointments that can fall under medical billing benefits or urgent medical care [17]. Importantly, some difficult or unusual oART cases may require full MDT input and, certainly in the future, AI may provide even faster solutions [7, 24].

The transition to an RT‐led oART model presents several challenges, primarily the additional training required to equip RTs with the necessary skills, knowledge, and clinical judgement capabilities to adapt treatment plans accurately [13]. The cost of the training and RT manpower required to deliver an RT‐led service has yet to be determined. It is worth noting that the costs for RT training in MRI adaptive radiotherapy will be higher due to the additional safety procedures and image interpretation involved [21] compared to CBCT‐guided oART. If we assess some of the published training frameworks for RT‐led oART, financial values can be assigned to each of the additional requirements recognising that RT‐led oART represents an extension to the current RT scope of practice following adequate training and credentialing [13]. These costs can be cross‐referenced with the value they potentially bring in facilitating the benefits of oART on patient outcomes such as toxicity reductions in reducing patient side effects and hypo‐fractionation improving patient convenience and satisfaction [24].

Further to this, the RT‐led model fits well within global and local advanced practice initiatives [28, 29] including the Australian Society of Medical Imaging and Radiation Therapy (ASMIRT) framework of an appropriate higher education degree [30]. Globally, there are some differences in training for RT‐led oART [13, 24]. In addition, there may be concerns about the scope of practice in RT‐led oART, as the expanded RT role may overlap with the responsibilities traditionally held by other members of the MDT [23]. Clear guidelines and protocols must be established to delineate roles and ensure efficient and collaborative practice [15]. For the MDT to function well during oART, it is necessary to have agreement between each of the professions on all aspects of the RT‐led oART workflow. In some institutions, agreement may not be reached, with an MDT‐led approach when treating with oART [14]. However, there is a growing body of evidence that demonstrates RTs are capable of leading oART to great accuracy and effectiveness, empowering the profession into the future alongside technological advances and the value they bring [23, 24, 25, 26].

Maintaining high standards of care is paramount in the RT‐led oART model, given the risk associated with altering the daily patient contours and treatment plan. The evidence base is limited on the rigour and cost associated with the QA processes in RT‐led oART. More broadly, peer review systems have been cited [18], facilitating ongoing professional development, and close collaboration with healthcare professionals to uphold treatment accuracy and patient safety [31]. Despite these challenges, the RT‐led oART versus an RO‐led or MDT model of care holds promise for enhancing efficiency, improved use of manpower (cost implications), cost‐effectiveness, and adaptability of radiotherapy teams to respond to innovation in the field and bring value to patients. Importantly, when changes are implemented, VBHC data should be evaluated to include the benefit of the change and improvements to treatment delivery, patient side effects, and quality of life [28]. While the RT‐led oART model will also likely result in improved patient outcomes, other VBHC metrics such as patient satisfaction and RT job satisfaction should be assessed concurrently [13, 20].

## 4 Staff Training and Satisfaction

The transition to a RT‐led oART model necessitates significant training, which can be both a challenge and an opportunity for professional growth [23, 24, 26]. The additional training costs for RTs for RT‐led oART have not been explored in the literature; however, many frameworks for training and credentialing have been published [13, 21, 23, 32]. Shepherd et al. [13], reported that RTs experienced increased job satisfaction due to their expanded roles in oART and greater involvement in applying technology to patient care. This is similar in nursing, where there may be concerns about increased responsibilities and the need for continuous education, leadership, and clinical experience [33]. Moreover, despite the concept of VBHC not being fully explored for RT‐led oART, Joyce et al. [34] indicated RTs are highly aware and value the impact of oART and the training it will bring in professional development opportunities to the profession.

Regarding satisfaction, on both fronts, traditional MDT models with the division of tasks among specialists can lead to good levels of job satisfaction. This is due to the clear delineation of roles and collaborative environment which it encourages [35]. The only drawback is that the need for constant coordination and communication can sometimes result in workflow inefficiencies and stress [34]. In contrast, the literature demonstrates the development of RTs in oART towards an advanced career pathway, embracing new technology, that can attract talent and promote higher retention within the profession and lifelong learning [35].

## 5 Patient Knowledge, Preferences and Shared Decision Making

Patient experience is a crucial determinant of the success of any healthcare model. The traditional MDT approach offers the advantage of comprehensive care with input from multiple specialists, which can enhance patient experience, confidence and potentially satisfaction. However, radiotherapy generally is poorly understood by patients [36]. Therefore, the complexity of oART may well require more consultation with consumers and focused efforts on generating patient understanding, prior to oART satisfaction. In addition, potential extended treatment times due to delays in online treatment planning may negatively impact the overall patient experience of oART, whether RT‐led or not.

There is limited published data on patient satisfaction, or knowledge and understanding of oART, let alone the RT‐led model. This is a broad scope gap in the literature and should be addressed in the future by oART teams to ensure that the consumer voice is heard. Patients have unique insights and may appreciate the quicker adjustments, fewer treatment interventions (i.e., rescanning, bladder or bowel filling and emptying) and potentially overall shorter treatment durations enabled by hypo fractionated oART. However, these benefits may initially come with longer individual treatment fractions due to the adaptive workflow, and the tradeoffs between extended session times and fewer total visits may not be acceptable or preferable to all patients. Understanding individual preferences and clearly communicating the benefits and tradeoffs is necessary to support shared decision making. The patient's close interaction with RTs can improve communication and personalised care; however, ensuring that patients understand and trust the new model is essential for maintaining high levels of satisfaction.

## 6 Future Directions and Policy Considerations for RT‐Led oART Models

Future research should focus on hybrid models that integrate the strengths of RT‐led workflows, traditional MDT oversight, and AI‐supported decision tools. In this context, ‘hybrid’ refers to models where trained RTs lead the adaptive process with support from advanced decision support systems, while maintaining access to specialist oversight (e.g., ROs or APRTs) when needed. This approach could enhance RT capability, optimise workflow efficiency, and uphold patient safety. Studies should also explore QA practices, long‐term patient outcomes [4], satisfaction, and cost‐effectiveness to fully evaluate the clinical value of these integrated models. Efforts to systematically capture the costs and benefits associated with oART practices are crucial in ensuring that rigorous analysis and evaluation can be conducted in the future. Only analysis of substantial datasets will allow for the profession to demonstrate that oART practices, regardless of staffing model, are cost‐effective.

From a policy perspective, it is crucial to establish clear guidelines and standards for the implementation of RT‐led oART models. Documentation and publication of successful RT‐led oART services will serve as a basis for advocating for professional recognition of RTs delivering adaptive treatments without an RO present. This is particularly important as the profession seeks to be recognised and licensed by state and federal regulatory bodies for RT‐led oART.

## 7 Conclusion

This commentary has evaluated the merits of conventional MDT approaches and RT‐led oART in the context of VBHC. The traditional MDT model, with its comprehensive expertise and collaborative decision‐making, ensures high quality patient outcomes but is often hindered by coordination challenges and higher costs. Conversely, the RT‐led oART model is likely to offer increased efficiency, cost‐effectiveness and timely treatment adjustments, although these claims are still speculative and require further research, especially given that this workflow is new and novel. While some costs, such as training, may be incurred upfront, we expect that oART processes will become more streamlined over time, particularly with the routine integration of AI across all workflows. Personalised medicine is becoming the mainstay of cancer treatments, and it is anticipated that adaptive radiotherapy treatments will become a more common technique used and may be preferred by patients.

The future of value‐based care in online adaptive radiotherapy lies in integrating the strengths of both models, including ones supported by AI. Hybrid approaches that leverage advanced decision‐support systems can enhance the capabilities of RTs while maintaining the benefits of specialist oversight, including APRTs. Continuous innovation, robust training programmes, and supportive policy frameworks and documentation will be crucial to these hybrid models, RT professional longevity, and improving patient outcomes.

In conclusion, both the MDT and RT‐led models have distinct advantages that contribute to the advancement of VBHC in oART. By fostering ongoing innovation, rigorous evaluation, and effective policy support, the radiation oncology community can achieve more efficient, cost‐effective, and patient‐centred treatment, ultimately enhancing the quality of care for patients.

## Conflicts of Interest

The authors declare no conflicts of interest.

## Data Availability

Data sharing not applicable to this article as no datasets were generated or analysed during the current study.
